# Activity-based labelling of ammonia- and alkane-oxidizing microorganisms including ammonia-oxidizing archaea

**DOI:** 10.1093/ismeco/ycae092

**Published:** 2024-07-11

**Authors:** Dimitra Sakoula, Arne Schatteman, Pieter Blom, Mike S M Jetten, Maartje A H J van Kessel, Laura Lehtovirta-Morley, Sebastian Lücker

**Affiliations:** Department of Microbiology, RIBES, Radboud University, Heyendaalseweg 135, 6525 AJ, Nijmegen, the Netherlands; School of Biological Sciences, University of East Anglia, Norwich Research Park, Norwich, Norfolk, NR4 7TJ, United Kingdom; Department of Microbiology, RIBES, Radboud University, Heyendaalseweg 135, 6525 AJ, Nijmegen, the Netherlands; Department of Microbiology, RIBES, Radboud University, Heyendaalseweg 135, 6525 AJ, Nijmegen, the Netherlands; Department of Microbiology, RIBES, Radboud University, Heyendaalseweg 135, 6525 AJ, Nijmegen, the Netherlands; School of Biological Sciences, University of East Anglia, Norwich Research Park, Norwich, Norfolk, NR4 7TJ, United Kingdom; Department of Microbiology, RIBES, Radboud University, Heyendaalseweg 135, 6525 AJ, Nijmegen, the Netherlands

**Keywords:** Ammonia-oxidizing bacteria, ammonia-oxidizing archaea, alkane-oxidizing bacteria, ammonia monooxygenase, methane monooxygenase, activity-based enzyme labelling, click chemistry, bifunctional enzyme probe

## Abstract

Recently, an activity-based labelling protocol for the in vivo detection of ammonia- and alkane-oxidizing bacteria became available. This functional tagging technique enabled targeted studies of these environmentally widespread functional groups, but it failed to capture ammonia-oxidizing archaea (AOA). Since their first discovery, AOA have emerged as key players within the biogeochemical nitrogen cycle, but our knowledge regarding their distribution and abundance in natural and engineered ecosystems is mainly derived from PCR-based and metagenomic studies. Furthermore, the archaeal ammonia monooxygenase is distinctly different from its bacterial counterparts and remains poorly understood. Here, we report on the development of an activity-based labelling protocol for the fluorescent detection of all ammonia- and alkane-oxidizing prokaryotes, including AOA. In this protocol, 1,5-hexadiyne is used as inhibitor of ammonia and alkane oxidation and as bifunctional enzyme probe for the fluorescent labelling of cells *via* the Cu(I)-catalyzed alkyne-azide cycloaddition reaction. Besides efficient activity-based labelling of ammonia- and alkane-oxidizing microorganisms, this method can also be employed in combination with deconvolution microscopy for determining the subcellular localization of their ammonia- and alkane-oxidizing enzyme systems. Labelling of these enzymes in diverse ammonia- and alkane-oxidizing microorganisms allowed their visualization on the cytoplasmic membranes, the intracytoplasmic membrane stacks of ammonia- and methane-oxidizing bacteria, and, fascinatingly, on vesicle-like structures in one AOA species. The development of this novel activity-based labelling method for ammonia- and alkane-oxidizers will be a valuable addition to the expanding molecular toolbox available for research of nitrifying and alkane-oxidizing microorganisms.

## Introduction

The oxidations of ammonia, methane, or short-chain alkanes are environmentally widespread processes catalyzed by a diverse range of microorganisms, which besides their fundamental role in the environment also are of large interest for biotechnological applications, e.g., for the treatment of wastewater and drinking water. Aerobic ammonia oxidation, the initial and rate-limiting step of the nitrification process, plays a key role in the biogeochemical nitrogen cycle and is catalyzed by chemolithoautotrophic ammonia-oxidizing prokaryotes. For decades, the metabolic potential to perform aerobic ammonia oxidation to nitrite had been considered a trait restricted to a few bacterial genera (ammonia-oxidizing bacteria; AOB). However, the discovery that archaea affiliated with the *Thaumarchaeota* phylum can also grow by oxidizing ammonia has fundamentally changed this perception [1] and the presence of ammonia-oxidizing archaea (AOA) has been verified in many ecologically diverse environments [2–6].

AOA are important microorganisms in aquatic ecosystems, where they can account for up to 40% of all prokaryotes. AOA also play a major role in nitrogen flux in terrestrial habitats, particularly acidic and natural soils [3, 7, 8]. So far, the detection of AOA in the environment has been largely based on the utilization of cultivation-independent sequencing-based techniques, like PCR and metagenomics [4]. These approaches have provided invaluable data regarding the distribution and abundance of AOA in a variety of environments but cannot directly detect their ammonia-oxidizing activity. The key enzyme of ammonia oxidation is ammonia monooxygenase (AMO), which catalyzes the oxygen-dependent conversion of ammonia to hydroxylamine. AMO enzymes belong to the copper-containing membrane monooxygenase (CuMMO) family, members of which catalyze a wide array of reactions, such as ammonia, methane, and short-chain hydrocarbon oxidation, and exhibit a high degree of genetic, structural, and catalytic similarities [9–11]. Also, the archaeal and bacterial AMO enzymes exhibit genetic and structural similarities that indicate a common evolutionary history [6]. However, it has been demonstrated that there are substantial dissimilarities regarding substrate range and catalytic properties [12]. For example, there are distinct differences in their sensitivity to the ammonia oxidation inhibitors allylthiourea (ATU) [13–15] and terminal n-alkynes [12]. Furthermore, the subunit composition of the archaeal ammonia monooxygenase is notably different from the bacterial members of the CuMMO superfamily. For instance, the AMO from the AOA *Nitrososphaera viennensis* contains six subunits instead of the three typically found in bacterial CuMMOs [16]. In addition, the archaeal AMO shares a relatively low sequence similarity (40%) to bacterial CuMMOs, and the bacterial CuMMOs, despite performing different primary functions like methane and ammonia oxidation, share a greater sequence similarity to each other than with the archaeal enzyme. AOA also lack intracellular membrane stacks that harbor the AMO enzyme in bacterial ammonia oxidizers. Furthermore, co-oxidation of a wide range of substrates other than ammonia is known in bacterial AMOs, but this topic remains underexplored in AOA [17]. Having an activity-based probe for the archaeal AMO would be extremely useful for addressing the knowledge gaps in the function, cellular localization, and environmental activity of this important enzyme and exploring its role in the success and adaptation of AOA in the environment.

An activity-based labelling method enabling the in vivo detection of CuMMO-containing bacteria in complex microbial communities has lately been developed [18, 19]. In this method, fluorescent labelling of CuMMO-containing bacteria, including the recently discovered complete ammonia-oxidizing (comammox) *Nitrospira* [20, 21], was achieved by the use of the irreversible CuMMO inactivator 1,7-octadiyne (1,7OD) in combination with the highly specific copper-catalyzed alkyne-azide cycloaddition (CuAAC) reaction. Despite the structural similarities between the bacterial and archaeal AMOs, however, ammonia oxidation in AOA has been shown to be insensitive to long-chain alkynes, such as 1-octyne [22] and, consequently, 1,7OD was unable to label AOA. Still, since the archaeal AMO is known to be sensitive to inactivation by shorter-chain terminal n-alkynes (<C_6_) [12, 22, 23], their corresponding diynes are promising candidates for bifunctional enzyme probes to also label AOA.

In this study, we therefore set out to investigate the suitability of 1,5-Hexadiyne (1,5HD), the shortest commercially available diyne, to be employed as an enzyme inactivator for the activity-based fluorescent labelling of all ammonia, methane, and short-chain alkane oxidizers, including AOA. In addition, we use this activity-based fluorescent labelling method to investigate the subcellular localization of the respective CuMMO enzymes in phylogenetically diverse ammonia and methane oxidizers.

## Materials and methods

### Cultivation

Pure cultures of *Nitrosarchaeum koreense* MY1 (Group 1.1a), *Nitrosocosmicus franklandus* (Group 1.1b), *Nitrosomonas europaea* (ATCC 25978) and *Methylotetracoccus oryzae* were grown as described before [24–27]. Highly enriched cultures of *Ca*. Nitrosotenuis chungbukensis MY2 [28] and *Ca*. Nitrospira kreftii were cultivated as described elsewhere [29]. The pure cultures of the propane-oxidizing *Rhodococcus sp.* strain ZPP and the butane-degrading *Thauera butanivorans* strain Bu-B1211 (DSM 2080) were maintained as described in Sakoula et al. [18]. *Escherichia coli* (DSMZ 498) cells were grown in LB medium (10 g L^−1^ NaCl, 5 g L^−1^ yeast extract, 10 g L^−1^ peptone), at 37°C, 150 rpm. Unless stated otherwise, all cultures were harvested in the late exponential phase. AOA were harvested by filtration using Amicon Ultra-15 filters (10 kDa cutoff; Millipore, Burlington, MA, USA). The filters were equilibrated by washing twice with sterile medium prior to use. Biomass from the remaining strains was harvested by gentle centrifugation (2000 × *g*, 15 min), washed twice with the respective sterile medium, and resuspended in sterile medium to a final density of approximately 100 μg total cell protein ml^−1^.

### Activity assays

Biomass was resuspended in the respective HEPES buffered (10 ml l^−1^ HEPES solution, consisting of 1 M HEPES and 0.6 M NaOH; pH 7.6) mineral salts medium and incubated in glass vials sealed with butyl rubber stoppers and aluminium crimp seals. Cells were incubated in the presence of 100 μΜ 1,5HD (aqueous concentration; 50% in pentane; Alfa Aesar, Haverhill, MA, USA) with either ammonium (1 mM for AOA and AOB, 0.12 mM for comammox), hydroxylamine (0.2 mM), or nitrite (0.1 mM), and methane (15% (v/v)) or methanol (0.15 mM) as substrates for ammonia- and alkane-oxidizing microorganisms, respectively. 1,5HD is reactive and unstable in the presence of oxygen and the commercial preparations contain an argon atmosphere. To avoid spontaneous breakdown, which could potentially interfere with the activity assays and labelling, the commercial stock of 1,5HD was aliquoted in an anoxic cabinet after opening, and aliquots were stored under N_2_ atmosphere until further use. Incubations with the same substrates with and without pentane addition (100 μΜ final concentration) but in the absence of 1,5HD were performed as controls. Abiotic hydroxylamine conversion was tested using sterile HEPES buffered pH 7.6 medium supplemented with 200 μΜ hydroxylamine (Fig. S1). Bottles were incubated with an ambient oxygen-containing atmosphere at the optimal growth temperature of each microorganism, in the dark without shaking. All incubations were performed in three biological replicates. Liquid samples (0.5 mL) were taken for the determination of ammonium, hydroxylamine, nitrite, nitrate, and methanol concentrations, as well as headspace gas samples (50 μl) for measuring methane, propane, and butane.

### Analytical methods

Ammonium was measured fluorometrically using a modified orthophatal-dialdehyde assay [30]. Nitrite and nitrate were measured colorimetrically by the Griess reaction [31] in combination with nitrate reduction by vanadium(III) [32], and hydroxylamine with 8-quinolinol [33]. Methane, propane, and butane concentrations in the headspace were measured using gas chromatography (HP 5890a, equipped with a flame ionization detector and a Porapak Q column at 80°C). Methanol was measured colorimetrically according to Mangos and Haas [34]. For the determination of protein content, cells were lysed with the Bacterial Protein Extraction Reagent (B-PER; Thermo Fisher Scientific, Waltham, MA, USA) according to manufacturer’s instructions in combination with mild sonication (1 minute, 30 Hz). Protein concentrations were measured using the Pierce bicinchoninic acid protein assay kit (Thermo Fisher Scientific, Waltham, MA, USA) according to the “Enhanced Test Tube” protocol. For all fluorometric and colorimetric measurements a Spark M10 plate reader (Tecan Trading AG, Männedorf, Switzerland) was used.

### In vivo activity-based labelling

Active biomass was harvested as described above, washed twice, and resuspended in 50 ml medium without substrates. Subsequently, the biomass was incubated with 100 μM 1,5HD for 30 minutes in the dark without shaking, at the optimal temperature of each culture. Following inactivation, cells were pelleted by centrifugation (2000 × *g*, 10 minutes), washed twice in sterile PBS, pH 7.4, and fixed using a 50% (v/v) ethanol in PBS solution for 10 min at ambient temperature (RT). Subsequently, the CuAAC reaction was performed in plastic microcentrifuge tubes (1.5 mL) in a final volume of 250 μL to label 1,5HD-inhibited cells. For this, fixed biomass was washed with PBS, resuspended in 221 μl PBS and mixed with 12.5 μl of a freshly prepared 100 mΜ sodium ascorbate solution (99% purity, Merck KGaA, Darmstadt, Germany) and 12.5 μl of a freshly prepared 100 mM aminoguanidine hydrochloride solution (98% purity, Merck KGaA, Darmstadt, Germany). A dye mixture composed of 1.25 μl of a 20 mM CuSO_4_ solution (99.99% purity, Merck KGaA, Darmstadt, Germany), 1.25 μl of 100 mM Tris(3-hydroxypropyltriazolylmethyl)amine in ddH_2_O (THPTA; 95% purity, Merck KGaA, Darmstadt, Germany), and 0.3 μl of 5 mM Azide-Fluor 488 in DMSO (≥99% purity, Merck KGaA, Darmstadt, Germany) was prepared and left to react in the dark for 3 minutes. Subsequently, 2.8 μl dye mixture were added to the cell suspensions. The tubes were gently mixed and incubated for 60 min (RT, in the dark). CuAAC reactions were terminated by harvesting the cells by centrifugation (2000 × *g*, 10 minutes). Cell pellets were washed three times with PBS, resuspended in 1:1 (v/v) ethanol:PBS and stored at −20°C.

### Fluorescence and deconvolution microscopy

Prior to microscopic analysis, 10 μl activity-based labelled samples were dried onto microscope slides, dehydrated using an increasing ethanol series (50, 80, and 100%; 3 min each), and stained with 0.1 μg/ml DAPI in PBS (4′,6-diamidino-2-phenylindole dihydrochloride; Merck KGaA, Darmstadt, Germany) for 15 minutes. Subsequently, the slides were washed in MQ water, air-dried and embedded in Vectashield mounting solution (Vector Laboratories Inc., Burlingame, CA, USA). CuAAC (activity-based) and DAPI-conferred fluorescent signals were recorded using the HyD hybrid detectors of a Leica TCS Sp8x confocal laser microscope (CLSM; Leica Microsystems B.V., Amsterdam, the Netherlands) equipped with a 405 nm UV diode and a pulsed white-light laser. Images were recorded using a 100× oil immersion objective at a resolution of 1024 × 1024 pixels and 8-bit depth.

For detecting the subcellular localization of the AMO- and particulate methane monooxygenase (pMMO)-derived fluorescent signal, the HyVolution deconvolution module of the Huygens Essential Suite (Scientific Volume Imaging B.V., Hilversum, The Netherlands) was used. Fluorescent images were acquired using the HyD hybrid detectors of a Leica Sp8x CLSM, a 100× oil immersion objective, a 0.5 AU pinhole size, at 1024 × 1024 pixels resolution. Deconvolution was performed using the resolution-optimized algorithm of the HyVolution module.

## Results and discussion

### Inhibition of the archaeal ammonia oxidation by 1,5HD

AOA are strongly inhibited by low concentrations (10 μM) of short-chain alkynes (≤ C_5_), but tolerate much higher concentrations of longer-chain-length alkynes (≥ C_6_), including concentrations that can completely inhibit ammonia oxidation activity in AOB [12, 23]. Due to the contrasting inhibition profiles of these compounds in AOA and AOB, long-chain alkynes have been used in many ecological studies as differential inhibitors to distinguish bacterial and archaeal contributions to ammonia oxidation in soil [22, 35, 36].

**Figure 1:**
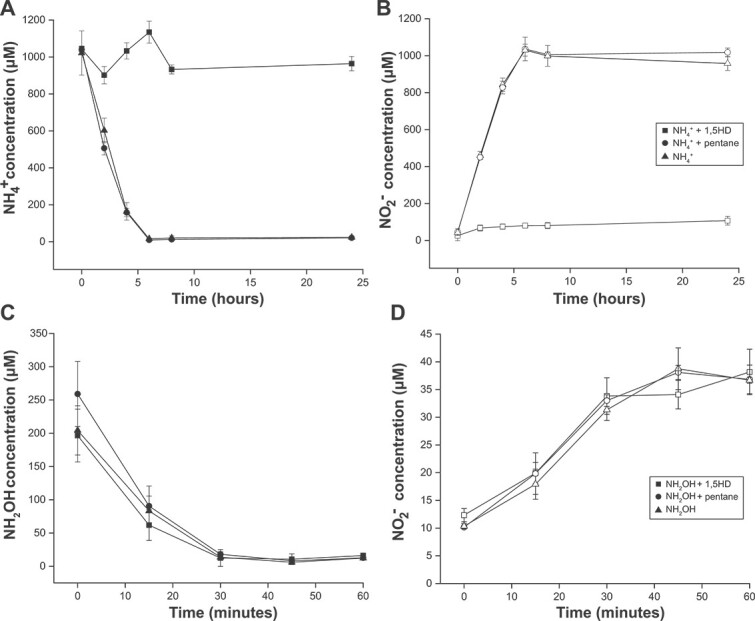
Effect of 1,5HD on ammonia oxidation by *Nitrosocosmicus franklandus*. (A) Ammonium consumption and (B) nitrite production in the presence of ammonium only (triangles), or with the addition of 1,5HD (squares) or pentane (circles). (C) Hydroxylamine consumption and (D) nitrite production in the presence of hydroxylamine only (triangles), or with 1,5HD (squares) or pentane (circles). Error bars represent standard deviations, calculated from three biological replicates.

In this study, we investigated whether the diyne counterparts to these alkynes displayed the same differential inhibition pattern in AOA and AOB. An earlier study [18] found that 1,7OD effectively inhibited ammonia and alkane oxidation in all tested bacterial strains, but was inefficient in binding to the archaeal AMO, which is consistent with previous reports that AOA tolerate much higher concentrations of 1-octyne than their bacterial counterparts and that the inhibition of AOA by 1-octyne is reversible [12, 22]. 1,5HD is the shortest chain-length diyne commercially available and was therefore chosen for characterization on a pure culture of *N. franklandus*. Previous studies found 1-hexyne to be less inhibitory to AOA than < C_5_ alkynes, but surprisingly, ammonia consumption by the AOA *N. franklandus* was completely inhibited by the addition of 100 μΜ 1,5HD, while 1 mM ammonium was stoichiometrically oxidized to nitrite in the absence of the inhibitor (Fig. 1A, B). No effect of 1,5HD on hydroxylamine oxidation was observed (Fig. 1C, D), suggesting a specific interaction of 1,5HD with the AMO enzyme. These results indicated that 1,5HD, at least at 100 μΜ concentration, is able to efficiently inhibit ammonia oxidation in archaea, and thus could potentially be used for activity-based labelling of AOA. In *Nitrosomonas europaea*, only small hydrocarbons (methane, acetylene) are competitive inhibitors of the AMO while larger hydrocarbons (>C_3_) act as non-competitive inhibitors [37]. This is consistent with what is known about AOA, as small hydrocarbons (methane, methanol, and acetylene) are competitive inhibitors of the archaeal AMO, but larger ones (phenylacetylene and octyne) are non-competitive [12, 38]. Although the mode of inhibition was not the focus of this study, based on previous studies the mode of inhibition by 1,5HD would be predicted to be non-competitive. Therefore, it is likely that the binding of 1,5HD to the CuMMOs would not be affected by changing or fluctuating environmental concentrations of ammonia.

### Inhibition of bacterial ammonia and alkane oxidation by 1,5HD

**Figure 2:**
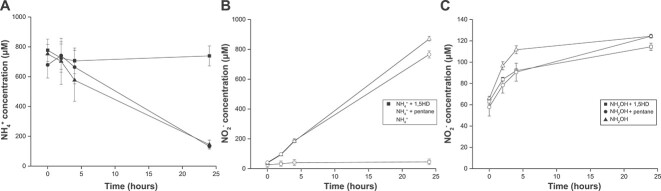
Inhibition of ammonia consumption by *Nitrosomonas europaea* by 1,5HD. (A) Ammonium consumption and (B) nitrite production in the presence of ammonium only (triangles), or with 1,5HD (squares) or pentane (circles). (C) Nitrite production by *N. europaea* in the presence of hydroxylamine only (triangles), or with 1,5HD (squares) or pentane (circles). Error bars represent standard deviations, calculated from three biological replicates.

To investigate the effect of 1,5HD on bacterial ammonia oxidation, we performed inhibition assays with a pure culture of *N. europaea*. Similar to *N. franklandus*, ammonia oxidation in *N. europaea* was completely inhibited by 100 μΜ 1,5HD (Fig. 2A, B). Again, hydroxylamine oxidation was not affected (Fig. 2C), indicating that 1,5HD specifically inhibits the AMO also in canonical AOB.

**Figure 3:**
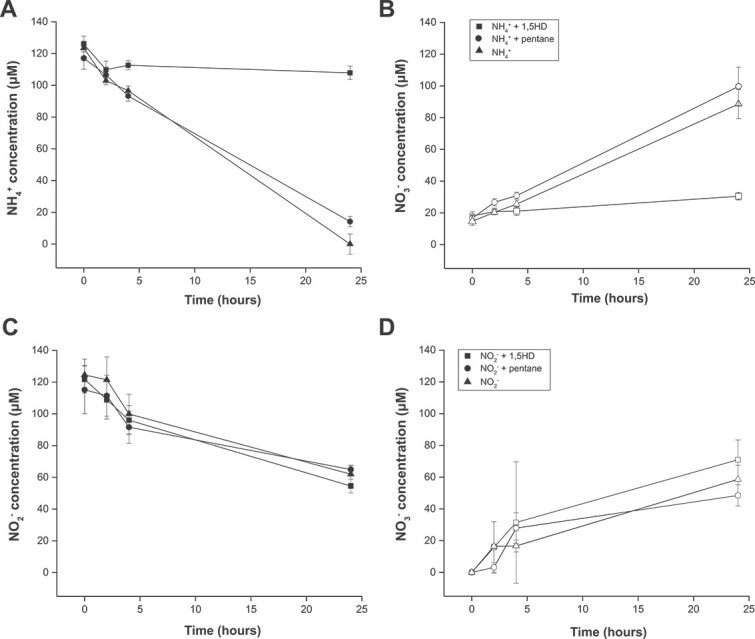
Inhibition of ammonia consumption by *Ca*. Nitrospira kreftii by 1,5HD. (A) Ammonium consumption and (B) nitrate production in the presence of ammonium only (triangles), or with 1,5HD (squares) or pentane (circles). (C) Nitrite consumption and (D) nitrate production in the presence of nitrite only (triangles), or with 1,5HD (squares) or pentane (circles). Error bars represent standard deviations, calculated from three biological replicates.

Comammox *Nitrospira* possess a phylogenetically distinct AMO [10, 20, 21, 29] and thus might react differently to ammonia oxidation inhibitors. To test the effect of 1,5HD on comammox bacteria, a highly enriched culture of *Ca*. N. kreftii was used. In the absence of the inhibitor, the culture stoichiometrically oxidized 120 μM ammonium to nitrate, whereas addition of 100 μΜ 1,5HD resulted in complete inhibition of ammonia oxidation (Fig. 3A, B). While the influence on hydroxylamine oxidation was not tested in this enrichment culture, no influence of 1,5HD on nitrite oxidation was observed (Fig. 3C, D), again showcasing that 1,5HD interacted with the AMO but not with the downstream pathway.

**Figure 4:**
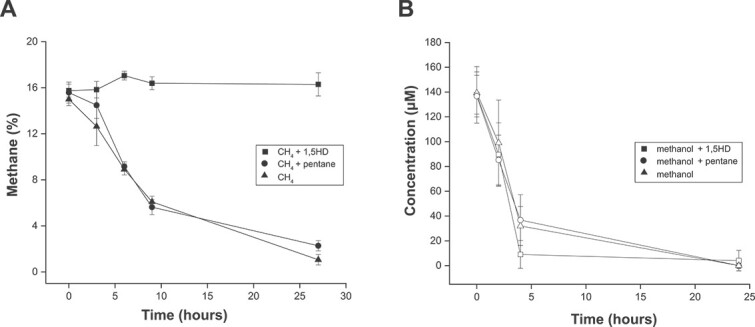
Inhibition of methane consumption by *Methylotetracoccus oryzae* by 1,5HD. (A) Methane consumption in the presence of methane only (triangles), or with 1,5HD (squares) or pentane (circles). (B) Methanol consumption in the presence of methanol only (triangles), or with 1,5HD (squares) or pentane (circles). Error bars represent standard deviation, calculated from three biological replicates.

Besides the ammonia monooxygenases, the CuMMO family includes enzymes that catalyze diverse reactions like the oxidation of alkanes such as methane and other short-chain (C_2_–C_4_) hydrocarbons, but still exhibit a high degree of structural similarity [9–11]. Thus, the ability of 1,5HD to inhibit methane-oxidizing bacteria was tested. Indeed, methane oxidation by the type Ib methanotroph *M. oryzae* was fully inhibited in the presence of 100 μΜ 1,5HD, while no effect on subsequent methanol oxidation was observed (Fig. 4). Thus, together with the successful labelling of additional ammonia- and alkane-oxidizing microorganisms (see below), our results indicate that 1,5HD can be employed as an inhibitor of CuMMO-containing microorganisms.

### In vivo activity-based fluorescent labelling of ammonia- and alkane-oxidizers

In *Nitrosomonas europaea*, the diyne 1,7OD irreversibly inactivates the AMO *via* a suicide inactivation mechanism [19] and has successfully been employed as a bifunctional enzyme probe that, in combination with a subsequent CuAAC reaction, allows the activity-based fluorescent staining of ammonia- and alkane-oxidizing bacteria [18]. However, AOA are only partly inhibited by octyne (≤ 40 μM) and this inhibition is fully reversible, indicating a different, non-covalent interaction with the archaeal AMO [12]. Consequently, activity-based staining of AOA cells was not possible using 1,7OD [18].

**Figure 5:**
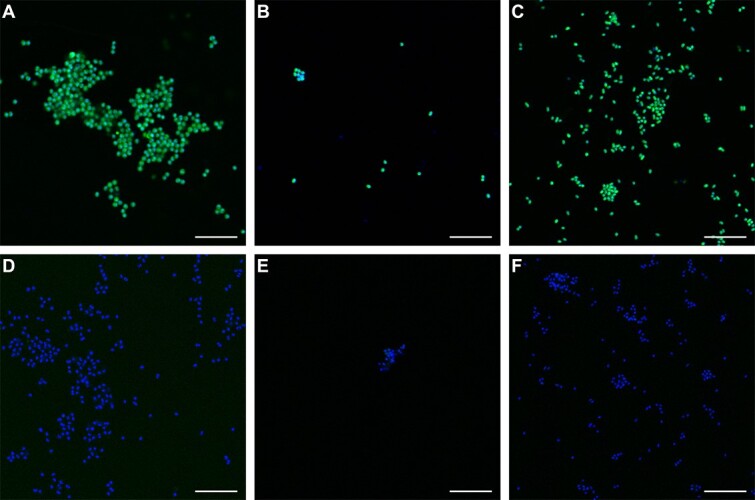
Activity-based fluorescent labelling of phylogenetically diverse AOA. (A, D) *N. franklandus*, (B, E) *Ca*. N. chungbukensis, and (C, F) *N. koreensis*. Cells were pre-incubated (A-C) in the presence and (D-F) without addition of 1,5HD. 1,5HD labelling is shown in green, DAPI staining in blue. Scale bars correspond to 10 μm.

In this study, we were able to demonstrate that the short-chain diyne 1,5HD efficiently inhibited ammonia oxidation in the AOA *N. franklandus*. Thus, its ability to be used for in vivo activity-based labelling of AOA was further investigated. For this, active cultures of *N. koreense*, *N. franklandus*, and *Ca*. N. chungbukensis were used. Following incubation with 1,5HD, ethanol fixation, and CuAAC reactions, all cultures were efficiently fluorescently stained (Fig. 5), indicating that 1,5HD can function as a bifunctional enzyme probe for the fluorescent labelling of phylogenetically diverse AOA. As the inhibition by 1,5HD did not interfere with hydroxylamine oxidation (Fig. 1), the observed fluorescence is likely due to specific binding of 1,5HD to the AMO enzyme, rather than alternative targets in the cell. The specific inhibition of CuMMOs by 1,5HD is further suggested by the observation that in the methanotroph *M. oryzae* 1,5HD inhibited methane oxidation, but not methanol oxidation. Additionally, the fact that the addition of 1,5HD is required for fluorescence suggests the binding of 1,5HD is covalent and irreversible. Considering that the covalent binding of acetylene to the AMO of *N. europaea* involves oxidation of acetylene to a ketene, it is likely that an oxidized derivative of 1,5HD, rather than 1,5HD itself, binds to the CuMMOs [39]. 1,5HD is only the third known irreversible inhibitor of the archaeal AMO besides acetylene and phenylacetylene [23]. Being able to fluorescently label active AOA will be a useful tool in environmental studies, as it will enable the identification of active members of mixed microbial communities based on monooxygenase enzyme activity.

**Figure 6:**
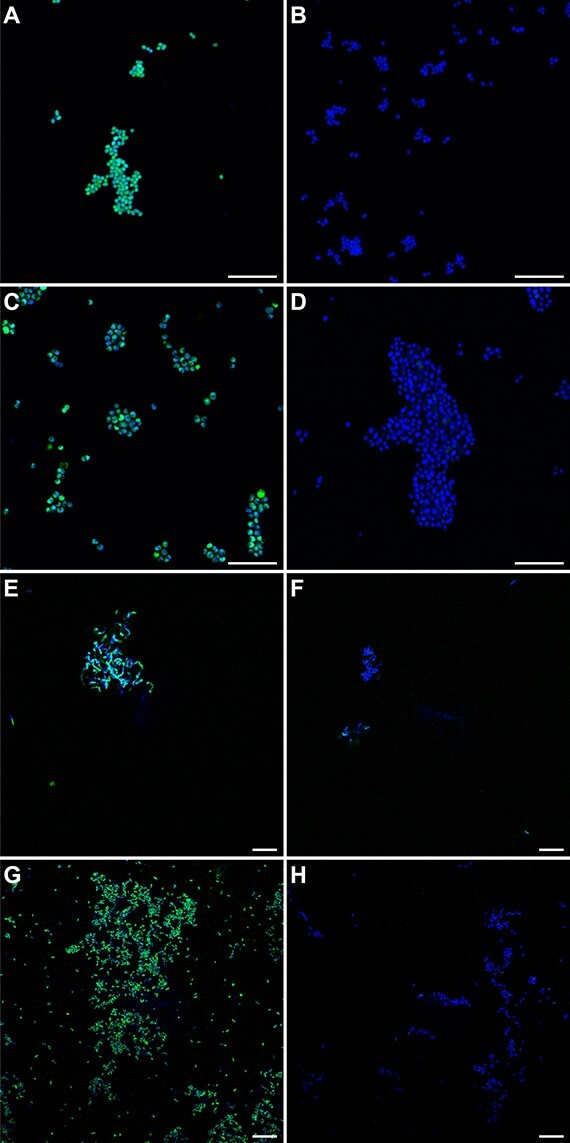
Activity-based fluorescent labelling of diverse ammonia- and alkane-oxidizing bacteria. (A, B) *N. europaea*, (C, D) *M. oryzae*, (E, F) *Rhodococcus sp.* ZPP, and (G, H) *Thauera butanivorans*. Cells were pre-incubated (A, C, E, G) in the presence and (B, D, F, H) without addition of 1,5HD. 1,5HD labelling is shown in green, DAPI staining in blue. Scale bars correspond to 10 μm.

The successful inhibition of ammonia- and alkane-oxidizing bacteria by 1,5HD (Figs. 2-4) also suggested interaction of 1,5HD with bacterial CuMMO and additionally with soluble di-iron monooxygenase (SDIMO) enzymes, as also has been observed for 1,7OD [18]. Consequently, when 1,5HD-treated biomass was subjected to the CuAAC protocol, strong fluorescent labelling was achieved for ammonia-oxidizing *N. europaea*, methane-oxidizing *M. oryzae*, propane-oxidizing *Rhodococcus* sp. ZPP, and butane-oxidizing *Thauera butanivorans* cells (Fig. 6). *Rhodococcus* sp. ZPP contains both a CuMMO and a SDIMO but was reported to use its CuMMO for the oxidation of propane [40], whereas *T. butanivorans* contains only a SDIMO [41]. In contrast, no background or unspecific labelling was observed when *E. coli* cells were subjected to the same protocol in a short incubation assay (Fig. S2). This efficient and specific labelling indicated that 1,5HD can be used as a probe for the function-based detection of CuMMO- and potentially also SDIMO-containing microorganisms. For instance, coupling activity-based probing with 1,5HD to fluorescence-activated cell sorting and sequencing-based approaches could yield novel insights into the ecology and metabolism of ammonia- and alkane-oxidizing microorganisms [18, 42, 43].

### Localization of the AMO enzyme in AOA

**Figure 7:**
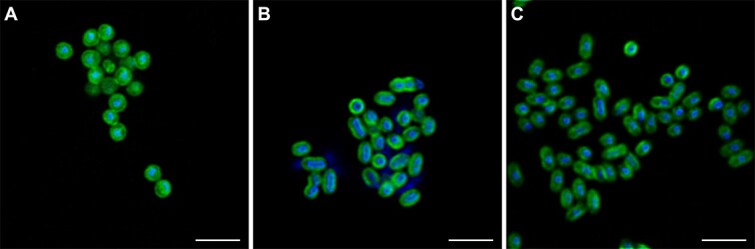
Subcellular localization of the AMO-derived fluorescent signal in AOA. (A) *N. franklandus*, (B) *Ca*. N. chungbukensis, and (C) *N. koreensis*. 1,5HD labelling is shown in green, DAPI staining in blue. Scale bars correspond to 2 μm.

**Figure 8:**
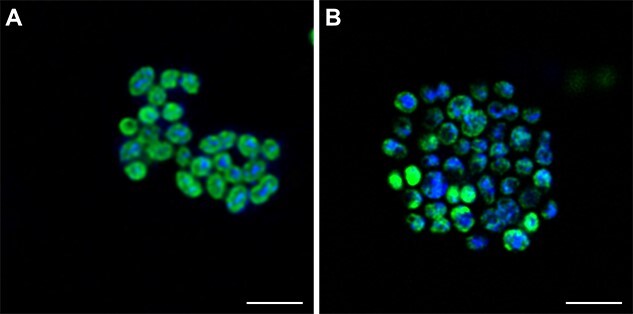
Subcellular localization of the AMO and pMMO-derived fluorescent signal in AOB and MOB, respectively. (A) *N. europaea* and (B) *M. oryzae*. 1,5HD labelling is shown in green, DAPI staining in blue. Scale bars correspond to 2 μm.

Like its bacterial homolog, the archaeal AMO enzyme is believed to reside on and be strongly associated with the cytoplasmic membrane [6], and fluorescent signals derived from AMO labelling are thus expected to colocalize with the cytoplasmic membrane. This assumption is supported by all characterized members of the CuMMO superfamily being membrane-bound [10], by the archaeal AMOs containing alpha helices predicted to span the membrane [16], by the native archaeal AMO complex being recovered from the membrane fraction of *N. viennensis* [16], and by the fact that AOA lack the membrane stacks found in AOB. Nevertheless, the subcellular localization of the archaeal AMO has not been explored previously. To visualize the subcellular AMO localization, three AOA cultures were subjected to the activity-based labelling protocol in combination with DAPI counter-staining. As expected, the localization of the AMO-derived signal was observed to coincide with the cytoplasmic membrane in high-resolution deconvolution micrographs in all cells (Fig. 7). Surprisingly, in addition to the plasma membrane in the periphery of the cells, some fluorescent labelling was evident in the middle of the cells of *N. franklandus* (Fig. 7A). This distribution of fluorescent signal in *N. franklandus* was more akin to bacterial ammonia oxidizers (Fig. 8) than with the other AOA tested in this study. While most AOA do not contain intracellular compartments or structures, *N. franklandus* has been described to have some intracellular compartmentalization, including vesicle-like structures of unknown function [44]. While fluorescence microscopy cannot conclusively prove that these vesicle-like structures harbor AMO, it is tempting to speculate based on the signal distribution we observed that they serve a similar function in increasing available membrane space available for ammonia oxidation as has been proposed for AOB. Alternatively, the distribution of the fluorescent label could be interpreted as concentrated patches of AMO on the plasma membrane. In this study, it was not possible to discern whether the fluorescent label was located inside the *N. franklandus* cells and on the vesicle-like structures, and the subcellular localization of the archaeal AMO should be further explored in future studies using, e.g., electron microscopy approaches.

Furthermore, the distribution of the fluorescent label in high-resolution deconvolution microscopy of the AOB *N. europaea* and the type Ib methanotroph *M. oryzae* suggests that the respective CuMMO-derived signals were localized along the intracytoplasmic membrane stacks and cytoplasmic membranes present in these organisms (Fig. 8), in agreement with previous studies showing the cytoplasmic membrane-associated localization of the AMO and pMMO enzymes [45, 46]. Still, the distribution of fluorophores might be affected by processing and cell damage, considering that the signal from DAPI is evident throughout the cell in some cases (Fig. 8). Thus, in addition to its usability for the targeted detection of ammonia- and alkane-oxidizing microorganisms in complex environmental samples, the activity-based CuMMO labelling method presented here also has great potential to study their cellular organization using high-resolution microscopy.

### Concluding remarks

Short-chain alkynes are effective inhibitors of ammonia oxidation in AOA, and we therefore hypothesized that short-chain diynes may be well suited for activity-based probing in AOA. The results presented here demonstrate that the diyne 1,5HD can be used to efficiently inhibit the activity of ammonia- and alkane-oxidizing microorganisms. Furthermore, we demonstrate that 1,5HD can be employed as a bifunctional enzyme probe for the activity-based fluorescent labelling of CuMMO- and certain SDIMO-containing cells. The development of this new labelling method for CuMMO-containing bacteria and archaea is an important advance as the existing 1,7OD-based method to label these organisms excluded AOA. As they are very abundant ammonia-oxidizers in diverse environments and crucial for nitrogen cycling, this imposed a significant limitation when studying active AOA populations. When combined with 16S rRNA-targeted fluorescence in situ hybridization (FISH), the activity-based labelling can be used for the phylogenetic identification of catalytically active ammonia- and alkane-oxidizing microorganisms present in complex microbial communities. Furthermore, this approach may be suitable for the retrieval of metabolically active cells from mixed populations. As such, it adds to the growing selection of activity-based methods such as BONCAT and ABPP with other probes, which can help to link phenotype and genotype. In addition, this method is a promising tool for the characterization of the subcellular localization of the archaeal AMO, which to date remains elusive. In conclusion, we are convinced that the development of this activity-based labelling method for ammonia- and alkane-oxidizers is a valuable addition to the molecular toolbox available to study these ubiquitous organisms with great environmental and biotechnological significance.

## Supplementary Material

5_Sakoula_et_al_Suplementary_material_ycae092

## Data Availability

The datasets generated and/or analyzed during the current study are available from the corresponding authors on request.

## References

[1] Könneke M , Bernhard AE, de la Torre JR et al. Isolation of an autotrophic ammonia-oxidizing marine archaeon. Natur*e* 2005;437:543–6. 10.1038/nature03911 16177789

[2] Herbold CW , Lehtovirta-Morley LE, Jung MY et al. Ammonia-oxidising archaea living at low pH: insights from comparative genomics. Environ Microbio*l* 2017;19:4939–52. 10.1111/1462-2920.13971 29098760 PMC5767755

[3] Lehtovirta-Morley LE , Stoecker K, Vilcinskas A et al. Cultivation of an obligate acidophilic ammonia oxidizer from a nitrifying acid soil. Proc Natl Acad Sci US*A* 2011;108:15892–7. 10.1073/pnas.1107196108 21896746 PMC3179093

[4] Prosser JI , Nicol GW. Relative contributions of archaea and bacteria to aerobic ammonia oxidation in the environment. Environ Microbio*l* 2008;10:2931–41. 10.1111/j.1462-2920.2008.01775.x 18973620

[5] Daebeler A , Herbold CW, Vierheilig J et al. Cultivation and genomic analysis of "*Nitrosocaldus islandicus*," an obligately thermophilic, ammonia-oxidizing Thaumarchaeon from a hot spring biofilm in Graendalur Valley. Iceland *Front Microbiol* 2018;9. 10.3389/fmicb.2018.00193 PMC5817080 29491853

[6] Schleper, C. and G.W. Nicol. Ammonia-oxidising archaea – Physiology, ecology and evolution. In: R.K. Poole (ed.), Advances in Microbial Physiolog*y*. Academic Press, 2010,1–41, 10.1016/B978-0-12-381045-8.00001-1. 21078440

[7] Karner MB , DeLong EF, Karl DM. Archaeal dominance in the mesopelagic zone of the Pacific Ocean. Natur*e* 2001;409:507–10. 10.1038/35054051 11206545

[8] Verhamme DT , Prosser JI, Nicol GW. Ammonia concentration determines differential growth of ammonia-oxidising archaea and bacteria in soil microcosms. ISME *J* 2011;5:1067–71. 10.1038/ismej.2010.191 21228892 PMC3131854

[9] Holmes AJ , Costello A, Lidstrom ME et al. Evidence that participate methane monooxygenase and ammonia monooxygenase may be evolutionarily related. FEMS Microbiol Let*t* 1995;132:203–8. 10.1111/j.1574-6968.1995.tb07834.x 7590173

[10] Khadka R , Clothier L, Wang L et al. Evolutionary history of copper membrane monooxygenases. Front Microbio*l* 2018;9:2493. 10.3389/fmicb.2018.02493 30420840 PMC6215863

[11] Rochman FF , Kwon M, Khadka R et al. Novel copper-containing membrane monooxygenases (CuMMOs) encoded by alkane-utilizing *Betaproteobacteria*. ISME *J* 2020;14:714–26. 10.1038/s41396-019-0561-2 31796935 PMC7031514

[12] Taylor AE , Taylor K, Tennigkeit B et al. Inhibitory effects of C_2_ to C_10_ 1-alkynes on ammonia oxidation in two *Nitrososphaera* species. Appl Environ Microbio*l* 2015;81:1942–8. 10.1128/AEM.03688-14 25576608 PMC4345366

[13] Hatzenpichler R , Lebedeva EV, Spieck E et al. A moderately thermophilic ammonia-oxidizing crenarchaeote from a hot spring. Proc Natl Acad Sci US*A* 2008;105:2134–9. 10.1073/pnas.0708857105 18250313 PMC2538889

[14] Lehtovirta-Morley LE , Verhamme DT, Nicol GW et al. Effect of nitrification inhibitors on the growth and activity of *Nitrosotalea devanaterra* in culture and soil. Soil Biol Bioche*m* 2013;62:129–33. 10.1016/j.soilbio.2013.01.020

[15] Shen TL , Stieglmeier M, Dai J et al. Responses of the terrestrial ammonia-oxidizing archaeon *Ca*. Nitrososphaera viennensis and the ammonia-oxidizing bacterium *Nitrosospira multiformis* to nitrification inhibitors. FEMS Microbiol Let*t* 2013;344:121–9. 10.1111/1574-6968.12164 23617238

[16] Hodgskiss LH , Melcher M, Kerou M et al. Unexpected complexity of the ammonia monooxygenase in archaea. ISME *J* 2023;17:588–99. 10.1038/s41396-023-01367-3 36721060 PMC10030591

[17] Rasche ME , Hyman MR, Arp DJ. Factors limiting aliphatic chlorocarbon degradation by *Nitrosomonas europaea*: cometabolic inactivation of ammonia monooxygenase and substrate specificity. Appl Environ Microbio*l* 1991;57:2986–94. 10.1128/aem.57.10.2986-2994.1991 16348568 PMC183909

[18] Sakoula D , Smith GJ, Frank J et al. Universal activity-based labeling method for ammonia- and alkane-oxidizing bacteria. ISME *J* 2022;16:958–71. 10.1038/s41396-021-01144-0 34743174 PMC8941013

[19] Bennett K , Sadler NC, Wright AT et al. Activity-based protein profiling of ammonia monooxygenase in *Nitrosomonas europaea*. Appl Environ Microbio*l* 2016;82:2270–9. 10.1128/AEM.03556-15 26826234 PMC4959501

[20] Daims H , Lebedeva EV, Pjevac P et al. Complete nitrification by *Nitrospira* bacteria. Natur*e* 2015;528:504–9. 10.1038/nature16461 26610024 PMC5152751

[21] van Kessel MAHJ , Speth DR, Albertsen M et al. Complete nitrification by a single microorganism. Natur*e* 2015;528:555–9. 10.1038/nature16459 26610025 PMC4878690

[22] Taylor AE , Vajrala N, Giguere AT et al. Use of aliphatic alkynes to discriminate soil nitrification activities of ammonia-oxidizing *Thaumarchaea* and *bacteria*. Appl Environ Microbio*l* 2013;79:6544–51. 10.1128/AEM.01928-13 23956393 PMC3811497

[23] Wright CL , Schatteman A, Crombie AT et al. Inhibition of ammonia monooxygenase from ammonia-oxidizing archaea by linear and aromatic alkynes. Appl Environ Microbio*l* 2020;86. 10.1128/AEM.02388-19 PMC7170481 32086308

[24] Jung MY , Park SJ, Kim SJ et al. A mesophilic, autotrophic, ammonia-oxidizing archaeon of thaumarchaeal group I.1a cultivated from a deep oligotrophic soil horizon. Appl Environ Microbio*l* 2014;80:3645–55. 10.1128/AEM.03730-13 24705324 PMC4054128

[25] Lehtovirta-Morley LE , Ross J, Hink L et al. Isolation of '*Nitrosocosmicus franklandus*', a novel ureolytic soil archaeal ammonia oxidiser with tolerance to high ammonia concentration. FEMS Microbiol Eco*l* 2016;92. 10.1093/femsec/fiw057 PMC4830249 26976843

[26] Koops H-P , Bottcher B, Moller UC et al. Classification of eight new species of ammonia-oxidizing bacteria: *Nitrosomonas communis* sp. nov., *Nitrosomonas ureae* sp. nov., *Nitrosomonas aestuarii* sp. nov., *Nitrosomonas marina* sp. nov., *Nitrosomonas nitrosa* sp. nov., *Nitrosomonas eutropha* sp. nov., *Nitrosomonas oligotropha* sp. nov. and *Nitrosomonas halophila* sp. nov. J Gen Microbio*l* 1991;137:1689–99. 10.1099/00221287-137-7-1689

[27] Ghashghavi M , Belova SE, Bodelier PLE et al. *Methylotetracoccus oryzae* strain C50C1 is a novel type Ib gammaproteobacterial methanotroph adapted to freshwater environments. mSpher*e* 2019;4:e00631–18. 10.1128/mSphere.00631-18 PMC6553558 31167950

[28] Jung MY , Park SJ, Min D et al. Enrichment and characterization of an autotrophic ammonia-oxidizing archaeon of mesophilic crenarchaeal group I.1a from an agricultural soil. Appl Environ Microbio*l* 2011;77:8635–47. 10.1128/AEM.05787-11 22003023 PMC3233086

[29] Sakoula D , Koch H, Frank J et al. Enrichment and physiological characterization of a novel comammox *Nitrospira* indicates ammonium inhibition of complete nitrification. ISME *J* 2021;15:1010–24. 10.1038/s41396-020-00827-4 33188298 PMC8115096

[30] Taylor S , Ninjoor V, Dowd DM et al. Cathepsin B2 measurement by sensitive fluorometric ammonia analysis. Anal Bioche*m* 1974;60:153–62. 10.1016/0003-2697(74)90140-7 4850914

[31] Griess P . Bemerkungen zu der Abhandlung der HH. Weselsky und Benedikt Ueber einige Azoverbindungen. Ber Dtsch Chem Ge*s* 1879;12:426–8. 10.1002/cber.187901201117

[32] Miranda KM , Espey MG, Wink DA. A rapid, simple spectrophotometric method for simultaneous detection of nitrate and nitrite. Nitric Oxid*e* 2001;5:62–71. 10.1006/niox.2000.0319 11178938

[33] Frear DS , Burrell RC. Spectrophotometric method for determining hydroxylamine reductase activity in higher plants. Anal Che*m* 1955;27:1664–5. 10.1021/ac60106a054

[34] Mangos TJ , Haas MJ. Enzymatic determination of methanol with alcohol oxidase, peroxidase, and the chromogen 2,2′-azinobis(3-ethylbenzthiazoline-6-sulfonic acid) and its application to the determination of the methyl ester content of pectins. J Agric Food Che*m* 1996;44:2977–81. 10.1021/jf960274z

[35] Ouyang Y , Norton JM, Stark JM et al. Ammonia-oxidizing bacteria are more responsive than archaea to nitrogen source in an agricultural soil. Soil Biol Bioche*m* 2016;96:4–15. 10.1016/j.soilbio.2016.01.012

[36] Lu XD , Bottomley PJ, Myrold DD. Contributions of ammonia-oxidizing archaea and bacteria to nitrification in Oregon forest soils. Soil Biol Bioche*m* 2015;85:54–62. 10.1016/j.soilbio.2015.02.034

[37] Keener WK , Arp DJ. Kinetic studies of ammonia monooxygenase inhibition in *Nitrosomonas europaea* by hydrocarbons and halogenated hydrocarbons in an optimized whole-cell assay. Appl Environ Microbio*l* 1993;59:2501–10. 10.1128/aem.59.8.2501-2510.1993 16349014 PMC182312

[38] Oudova-Rivera B , Wright CL, Crombie AT et al. The effect of methane and methanol on the terrestrial ammonia-oxidizing archaeon '*Nitrosocosmicus franklandus* C13'. Environ Microbio*l* 2023;25:948–61. 10.1111/1462-2920.16316 36598494

[39] Gilch S , Vogel M, Lorenz MW et al. Interaction of the mechanism-based inactivator acetylene with ammonia monooxygenase of *Nitrosomonas europaea*. Microbiolog*y* 2009;155:279–84. 10.1099/mic.0.023721-0 19118368

[40] Zou B , Huang Y, Zhang PP et al. Horizontal gene transfer of genes encoding copper-containing membrane-bound monooxygenase (CuMMO) and soluble di-iron monooxygenase (SDIMO) in ethane- and propane-oxidizing *Rhodococcus* bacteria. Appl Environ Microbio*l* 2021;87:e00227–1. 10.1128/AEM.00227-21 33962978 PMC8231442

[41] Dubbels BL , Sayavedra-Soto LA, Bottomley PJ et al. *Thauera butanivorans* sp. nov., a C_2_-C_9_ alkane-oxidizing bacterium previously referred to as *pseudomonas butanovora*. Int J Syst Evol Microbio*l* 2009;59:1576–8. 10.1099/ijs.0.000638-0 19528200 PMC2889399

[42] Couradeau E , Sasse J, Goudeau D et al. Probing the active fraction of soil microbiomes using BONCAT-FACS. *Nature*. Communication*s* 2019;10:10. 10.1038/s41467-019-10542-0 PMC6591230 31235780

[43] Ngara TR , Zhang HJ. Recent advances in function-based metagenomic screening. Genomics, Proteomics & Bioinformatic*s* 2018;16:405–15. 10.1016/j.gpb.2018.01.002 PMC6411959 30597257

[44] Klein T , Poghosyan L, Barclay JE et al. Cultivation of ammonia-oxidising archaea on solid medium. FEMS Microbiol Let*t* 2022;369. 10.1093/femsle/fnac029 PMC9072212 35323924

[45] Fiencke C , Bock E. Immunocytochemical localization of membrane-bound ammonia monooxygenase in cells of ammonia oxidizing bacteria. Arch Microbio*l* 2006;185:99–106. 10.1007/s00203-005-0074-4 16395553

[46] Kitmitto A , Myronova N, Basu P et al. Characterization and structural analysis of an active particulate methane monooxygenase trimer from *Methylococcus capsulatus* (bath). Biochemistr*y* 2005;44:10954–65. 10.1021/bi050820u 16101279

