# Supplementary material for: Activity-based labelling of ammonia- and alkane-oxidizing microorganisms including ammonia-oxidizing archaea
Source: ISME Commun. 2024 Jul 11;4(1):ycae092. doi: 10.1093/ismeco/ycae092 (PMC11283641; doi:10.1093/ismeco/ycae092)
Supplement: 5_Sakoula_et_al_Suplementary_material_ycae092 [file 5_sakoula_et_al_suplementary_material_ycae092.pdf]

Supplementary materials

Activity-based labelling of ammonia and alkane-oxidizing microorganisms including ammonia-oxidizing archaea

Running title: Activity-based labelling of ammonia- and alkane-oxidizers

Dimitra Sakoula[1#*], Arne Schatteman[2], Pieter Blom[1], Mike S.M. Jetten[1], Laura Lehtovirta-Morley[2], Maartje A.H.J. van Kessel[1], Sebastian Lücker[1*]

This PDF file includes:

Supplementary Figures S1 and S2

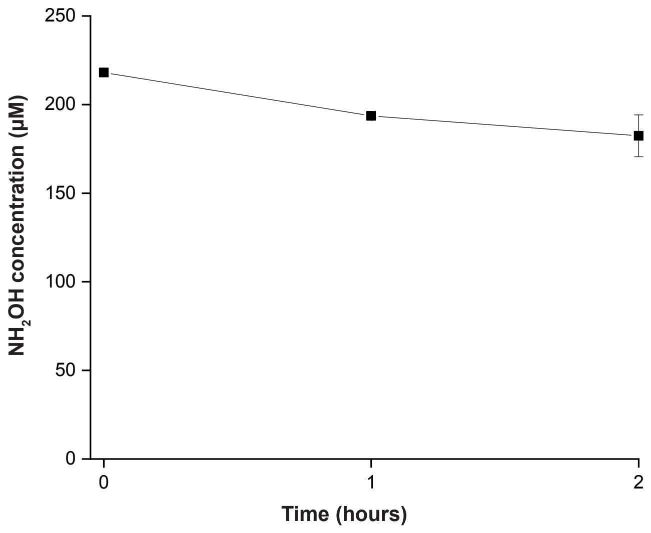

Supplementary Figure S1. Abiotic hydroxylamine oxidation in sterile mineral salt medium. Error bars represent standard deviations, calculated from three biological replicates.

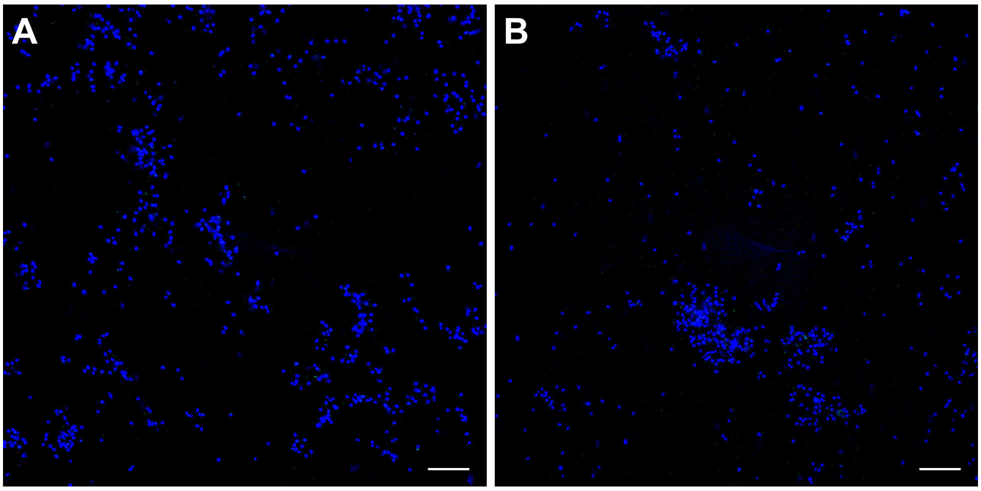

Supplementary Figure S2. Activity-based fluorescent labelling of active *E. coli* cells. Cells were pre-incubated (A) in the presence and (B) without addition of 1,5HD. CuMMO labelling is shown in green, DAPI staining in blue. Note the absence of CuMMO-derived signal, indicating the specificity of the activity-based labelling method. Scale bars correspond to 10 µm.
